# Myocardial tissue Doppler echocardiography and N-terminal B-type natriuretic peptide (NT-proBNP) in diastolic and systolic heart failure

**DOI:** 10.1186/1476-7120-6-45

**Published:** 2008-09-08

**Authors:** Fabian Knebel, Stephan Eddicks, Ingolf Schimke, Michael Bierbaum, Sebastian Schattke, Mark Beling, Vanessa Raab, Gert Baumann, Adrian C Borges

**Affiliations:** 1 Universitätsmedizin Berlin, Medical Clinic for Cardiology and Angiology, Charité Campus Mitte, Charitéplatz 1, 10098 Berlin, Germany; 2 Paritätisches Krankenhaus Lichtenberg, Fanningerstraß 32, 10365 Berlin, Germany; 3 Städtisches Klinikum München Schwabing, Kölner Platz 1, 80804 Munich, Germany

## Abstract

**Background:**

The aim of this prospective study was to assess the diagnostic value of NT-proBNP and the concordance with Tissue Doppler Echocardiography (including strain and longitudinal displacement) in diastolic and systolic heart failure.

**Methods and results:**

137 consecutive clinically stable patients were included (42 healthy controls, 43 with diastolic heart failure, 52 with systolic heart failure). In diastolic heart failure, basal septal strain was reduced (-24.8 ± 8.1% vs. controls. -18.5 ± 5.3%, p < 0.0001). In all patients with preserved systolic function, septal basal longitudinal displacement was impaired in patients with increased left-ventricular filling pressures (E/E' < 8: 13.5 mm ± 3.3 mm vs. E/E' > 15: 8.5 mm ± 2.3 mm, p = 0.001) parallel to NT-proBNP elevation (E/E' < 8: 45.8 pg/ml, IQR: 172.5 pg/ml vs. E/E' > 15: 402.0 pg/ml, IQR: 1337.2 pg/ml; p = 0.0007). In ROC analysis, NT-proBNP could detect patients with reduced left ventricular systolic function (LVEF ≥ 55%) with a good diagnostic accuracy. However, the diagnostic accuracy of NT-proBNP to detect diastolic dysfunction was lower.

**Conclusion:**

Subtle changes of longitudinal myocardial function begin in diastolic heart failure and are further increased in systolic heart failure. In patients with preserved LV function, a complex approach with the integration of multiple parameters including Tissue Doppler echocardiography and NT-proBNP is necessary to classify patients.

## Background

The prevalence of both systolic and diastolic heart failure is high and the prognosis is comparably poor. The prevalence of diastolic heart failure is increasing and the survival rates remain low, whereas the survival rates of systolic heart failure have improved in recent years. Diastolic heart failure is characterized by abnormal myocardial relaxation and increased passive stiffness and is hard to distinguish from systolic heart failure by clinical examination alone [1-8].

It was suggested that there is no isolated diastolic dysfunction, but that there is a continuum from normal to impaired diastolic and then systolic dysfunction (concept of "single syndrome"). The impairment of the longitudinal systolic function measured by Tissue Doppler Imaging (TDI) in patients with diastolic dysfunction supports this concept [9-11]. In contrast, the "two syndromes" concept claims two different pathological entities in systolic and diastolic heart failure. However, there is no final consensus on the taxonomy and the optimal diagnostic algorithm for the detection of diastolic dysfunction. Symptoms of heart failure, biomarkers, stress tests, and echocardiography are important elements in the diagnostic work up [12].

The non-invasive methods to diagnose heart failure include echocardiography and cardiac biomakers. Increased left-ventricular filling pressure is believed to induce myocardial wall-stress, the release of natriuretic peptides and increased E/E'-ratio. Diastolic heart failure is unlikely if E/E' is < 8, an E/E' 8–15 is suggestive of but not diagnostic of diastolic heart failure [13,14].

In addition to myocardial velocity measurements, new sensitive TDI-derived measurements of systolic function were introduced recently: Strain and longitudinal displacement [15]. Strain measures compression and distension of myocardial segments ("deformation imaging") and is reduced in systolic dysfunction [16,17]. Longitudinal displacement measures myocardial motion amplitudes in systole and visualizes the segmental velocity-time integrals. It correlates with longitudinal systolic function [18] but has not yet been examined in isolated diastolic dysfunction.

Natriuretic peptides (BNP and the hormonally inactive NT-proBNP) are significantly elevated in systolic and in less so in diastolic heart failure [19-22]. NT-proBNP correlates to prognosis in systolic heart failure [23,24], but its diagnostic value in stable asymptomatic patients with diastolic heart failure is still controversial.

The aim of this study was to assess the diagnostic value of NT-proBNP and the concordance with Tissue Doppler Echocardiography (Strain imaging, longitudinal displacement, E/E') in diastolic and systolic heart failure. We postulate that the integration of these parameters improves the severity estimation of systolic and diastolic heart failure.

## Methods

### Patients

In this prospective monocentric study (enrolment 2004–2005), 137 consecutive clinically stable out- and in-patients with a clinical indication for echocardiography from medical and surgical departments were included. Exclusion criteria: atrial fibrillation, relevant valvular heart disease exceeding mild mitral or aortic valve disease, prosthetic heart valves, pulmonary hypertension, myocardial infarction < 3 months prior to study inclusion, terminal renal failure, creatinine > 2.5 mg/dl, pregnancy, age < 18 years. The blood for NT-proBNP measurements (Elecsys proBNP, Roche Diagnostics, Germany [25] was drawn after echocardiography, centrifuged and frozen at -80°C immediately. The echocardiography examiners were blinded to the NT-proBNP values. The creatinine clearance was calculated as previously described [26,27].

Written informed consent was obtained from each patient. The ethics committee of the Charité University Hospital approved the protocol.

### Echocardiography

Transthoracic echocardiography was performed according to the ASE recommendations [28] by Vivid 7 Dimension (M3S 1.5–4.0 MHz transducer; GE Vingmed, Horton, Norway). The images were stored digitally and analyzed off-line by EchoPac PC Dimension (GE Vingmed, Horton Norway). Echocardiographic examinations included the trans-mitral inflow profile (E/A), TDI measurement of the ratio of the early-to-late annular velocity (E'/A') in the basal septum and left lateral myocardium at the mitral annulus, the E/E' ratio using the average of the basal septal and basal lateral E', the systolic basal septal and lateral myocardial velocities (S') as well as basal septal and lateral Strain and longitudinal displacement. (For acquisition of the TDI images: see **additional file **1 and **additional file **2]. All measurements were performed in the apical four chamber view; three beats were stored and analyzed.

The LVEF was calculated according to Simpson's rule [29]. A normal LVEF was defined as ≥ 55%, 30–55% is mild-moderately abnormal, a LVEF < 30% is severely abnormal according to [30]. LV mass was computed according to the ASE cube method [31].

Diastolic heart failure was defined as previously described: normal LVEF (≥ 55%) [30], E/E' > 10 [13,14], E/A < 1 [32,33]. The transmital flow and TDI parameters were adjusted to age-related cut-points according to [33,34].

The patients were classified as normal controls **(group 1)**, diastolic heart failure with preserved left ventricular function (LVEF ≥ 55%, **group 2**), systolic heart failure (LVEF < 55%, **group 3**).

### Statistics

Statistics were calculated by SPSS (version 12.0, Chicago, Ill, USA). Descriptive statistics of parametric variables are expressed as mean (± SD). Nonparametric variables are expressed as median (inter-quartile range, IQR, of 25 and 75 percentiles).

The comparison of echocardiographic parameters between groups was calculated by Wilcoxon test for non-parametric data. The Dunnett test was used for comparison to normal findings [35]. Dichotomized data were analyzed by the Chi^2^-test. The level of significance was p = 0.05.

ROC (Receiver Operator Characteristics) analysis was performed to calculate sensitivity, specificity, negative and positive predictive values and an optimal cut-point of NT-proBNP to detect systolic or diastolic dysfunction. The optimal cut-off point was assessed according to Youden [36].

## Results

137 patients were included. 42 patients had normal systolic and diastolic function (group 1), 43 patients had diastolic dysfunction (group 2), 52 patients had systolic dysfunction with an EF < 55% (group 3).

**Figure 1:**
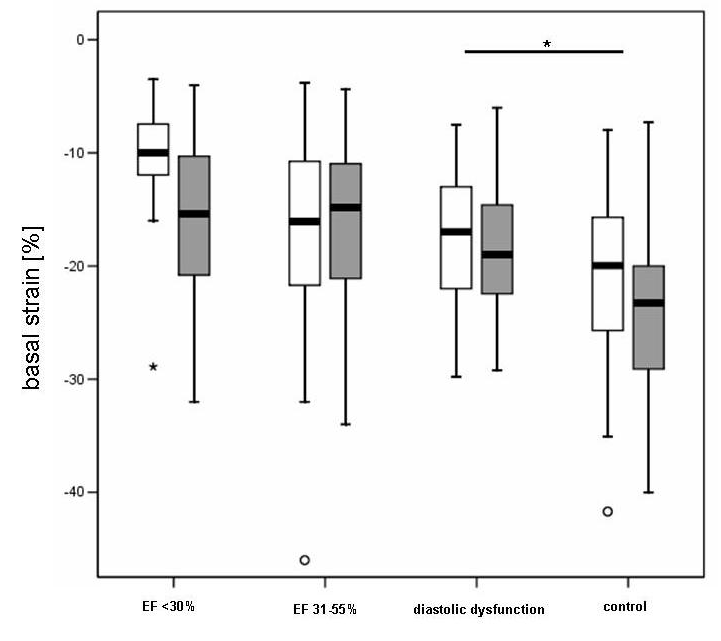
**Basal segment strain [%] in patients with severely and moderately reduced left ventricular function and patients with preserved systolic function and diastolic dysfunction**. Right boxplots: healthy controls. White: lateral, grey: septal strain.

The baseline characteristics of the patients are listed in table 1; the echocardiographic findings are listed in table 2. Patients with reduced LVEF (< 55%) had significantly increased NT-proBNP values compared to the healthy controls. Strain and longitudinal displacement parameters were significantly reduced in severely reduced LVEF compared to controls (table 3, figure 1).

**Table 1:** Baseline characteristics of the patients (mean ± SD, for non-parametric values: median, inter-quartile range) p compared to normal.

	**All patients (n= 137)**	Normal (n= 42)	Diastolic dysfunction (n = 43)	Systolic dysfunction (n = 52)	**p (compared to normal)**
Male sex (%)	**88 (65)**	23 (55)	23 (54)	43 (83)	1.00/0.0059
Age [y]	**53.8 (± 18.1)**	37.8 (± 15.9)	62.7 (± 12.5)	59.5 (± 15.2)	**< 0.0001/< 0.0001**
BMI [kg/m^2^]	**25.54 (± 4.36)**	23.8 (± 3.4)	26.5 (± 3.8)	26.2 (± 5.1)	**0.011/0.018**
NT-proBNP [pg/ml]	**2378 ± 6253, (median 222 (± 1220)**	275.9 ± 519.9 (median **66.8**, ± 185.3)	255.9 ± 137.4(median **137**, ± 256.7)	5832 ± 9185(median **1583**, ± 5109)	0.36/<**0.0001**
Creatinine clearance [ml/min]	**87.48 (± 37.89)**	107.9 (± 28.6)	82.1 (± 35.0)	78.5 (± 41.3)	**0.003/0.0002**
Heart rate [/s]	**71.6 (± 13.6)**	68.3 (± 13.9)	73.1 (± 13.1)	73.5 (± 13.5)	0.68/0.17
Systolic RR [mmHg]	**123.0**	123.0	136.8	115.4	**0.01**/0.19
Diastolic RR [mmHg]	**74.0**	75.4	80.0	71.2	0.21/0.23
coronary artery disease (%)	**41 (30)**	1 (2)	10 (23)	28 (54)	**0.0077/0.001**
previous myocardial infarction (%)	**28 (20)**	0	5 (12)	21 (40)	0.06/**0.0001**
arterial hypertension (%)	**63 (46)**	11 (26)	27 (63)	25 (48)	**0.0008/0.049**
diabetes mellitus (%)	**25 (18)**	3 (7)	3 (7)	18 (35)	1.00/**0.0046**
Hyperlipidemia (%)	**40 (29)**	2 (5)	15 (35)	22 (42)	**0.0018/< 0.0001**
Smoker (%)	**27 (20)**	10 (24)	9 (21)	9 (17)	0.8035
ischemic cardiomyopathy (%)	**30 (22)**	0	0	29 (56)	< 0.0001

**Table 2:** Echocardiographic findings. Mean ± SD

	**Normal**	**Diastolic dysfunction**	**Systolic dysfunction**	**P (compared to normal)**
LVEF (%)	59.5 (± 2.2)	59.1 (± 1.9)	31.5 (± 9.9)	0.99/**< 0.0001**
Fractional shortening (%)	0.38 (± 0.1)	0.39 (± 0.1)	0.16 (± 0.1)	0.58/**< 0.0001**
LVEDD (mm)	46.1 (± 4.2)	46.8 (± 5.5)	64.5 (± 12.2)	0.91/**< 0.0001**
LVESD (mm)	28.6 (± 5.9)	46.4 (± 5.7)	52.1 (± 15.0)	0.99/**< 0.0001**
PAP (mmHg)	26.1 (± 11.4)	27.0 (± 5.8)	38.4 (± 13.2)	0.94/**0.0015**
Septum (mm)	10.3 (± 1.9)	12.0 (± 2.8)	11.5 (± 2.0)	0.02/**0.0007**
Posterior wall (mm)	10.2 (± 1.7)	11.7 (± 1.8)	11.7 (± 1.5)	**0.001/< 0.0001**
E/A transmitral	1.5 (± 0.5)	0.9 (± 0.2)	1.3 (± 0.8)	**< 0.0001**/0.36
Left ventricular mass [mg]	195.4 (± 59.5)	236.1 (± 58.3)	436.7 (175.9)	0.19/**< 0.0001**
LVMI	104.1 (± 26.6)	126.0 (± 28.7)	222.6 (± 84.8)	0.14/**< 0.0001**
Strain septal (%)	-24.8 (± 8.1)	-18.5 (± 5.3)	-16.1 (± 7.0)	**< 0.0001/< 0.0001**
Strain left lateral (%)	-21.9 (± 11.4)	-17.6 (± 6.0)	-14.1 (± 8.3)	**0.04/< 0.0001**
septal longitudinal displacement (mm)	12.9 (± 3.0)	11.8 (± 2.1)	6.7 (± 3.9)	0.19/**< 0.0001**
lateral longitudinal displacement (mm)	12.1 (± 3.4)	10.9 (± 2.9)	7.4 (± 4.0)	0.20/<**0.0001**
TVI velocity E septal (m/s)	0.09 (± 0.02)	0.05 (± 0.01)	0.04 (± 0.02)	**< 0.0001/< 0.0001**
TVI velocity A septal (m/s)	0.06 (± 0.02)	0.08 (± 0.02)	0.06 (± 0.08)	0.26/0.98
TVI velocity S septal (m/s)	0.06 (± 0.01)	0.06 (± 0.01)	0.04 (± 0.01)	0.14/**< 0.0001**
E/E'	9.14 (± 4.62)	11.44 (± 3.14)	20.56 (± 15.08)	0.44/<**0.0001**
E'/A'	1.94 (± 1.17)	0.88 (± 0.60)	1.35 (± 0.92)	**< 0.0001/0.01**

**Table 3:** NT-proBNP and Tissue Doppler echocardiography variables according to reduction in systolic function, n = 137. (median ± SD, IQR: inter quartile range for NT-proBNP)

	**LVEF > 55%**	**LVEF 30–54%**	**LVEF < 30%**	**p vs normal**
NT-proBNP	97.0 (180.5)	587.6 (2914.9)	3373.0 (6057)	**< 0.001/< 0.001**
septal longitudinal displacement [mm]	11.5 (± 3.2)	9.3 (± 4.3)	4.9 (± 2.0)	**0.015/< 0.001**
lateral longitudinal displacement [mm]	12.6 (± 2.6)	8.7 (± 3.6)	4.1 (± 3.3)	**< 0.001/< 0.001**
Strain septal [%]	-21.1 (± 7.5)	-14.8 (± 6.9)	-15.4 (± 7.4)	**0.001/0.002**
Strain lateral [%]	-19.1 (± 9.3)	-16.1 (± 9.0)	-10.0 (± 5.2)	0.130/**< 0.001**
TVI S' [m/s] septal	0.09 (± 0.07)	0.06 (± 0.03)	0.04 (± 0.02)	**0.002/< 0.001**
TVI S' [m/s] lateral	0.06 (± 0.03)	0.05 (± 0.02)	0.03 (± 0.02)	**< 0.001/< 0.001**
E/E'	9.5 (± 4.1)	12.3 (± 12.0)	19.3 (± 16.6)	0.063/**< 0.001**

**Figure 2:**
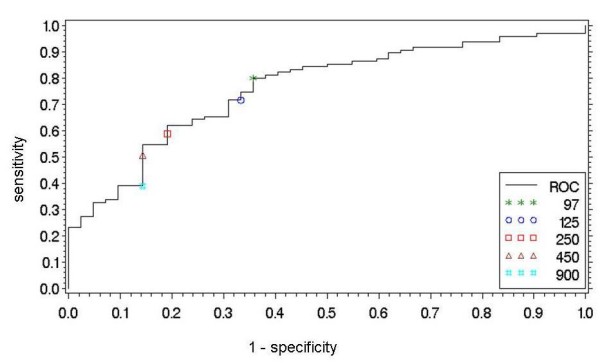
**Receiver Operating Characteristic curve to evaluate the diagnostic accuracy of NT-proBNP to separate patients with diastolic and/or systolic dysfunction (n = 95) from healthy controls (n = 42)**. The area under the curve (AUC) = 0.763 (p < 0.0001), Youden index = 0.44. The optimal cut-off is 97 pg/ml.

The ROC analysis to discriminate between normal LVEF (n = 88) and reduced LVEF (n = 49) had an area under the curve of 0.844, which indicates a good diagnostic accuracy. The best cut-off for this discrimination was 489 pg/ml (sensitivity 81.6%, specificity 85.2%, PPV 75.5% and NPV 89.3%, OR 25.6, Youden Index 0.67). The ROC analysis to discriminate between normal echocardiography (n = 42) and impaired diastolic and/or systolic function (n = 95) had an area under the curve of 0.763, which indicates a fair diagnostic accuracy. The best cut-off for this discrimination was 97 pg/ml (sensitivity 80.4%, specificity 64.3%, PPV 83.5% and NPV 58.7%, OR 7.2, Youden Index 0.44) (figure 2)

**Figure 3:**
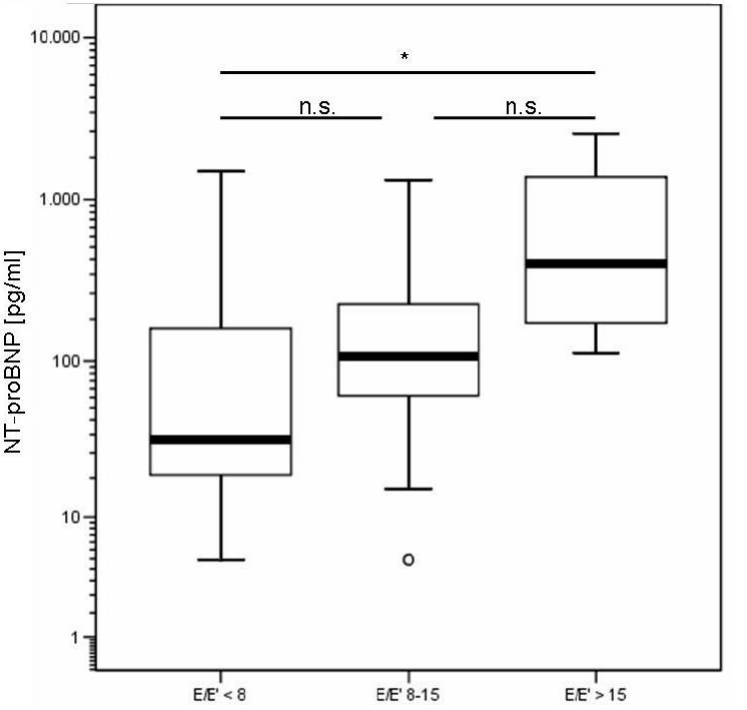
NT-proBNP [pg/ml] in patients with normal systolic function (n = 85) according to E/E' ratio.

**Figure 4:**
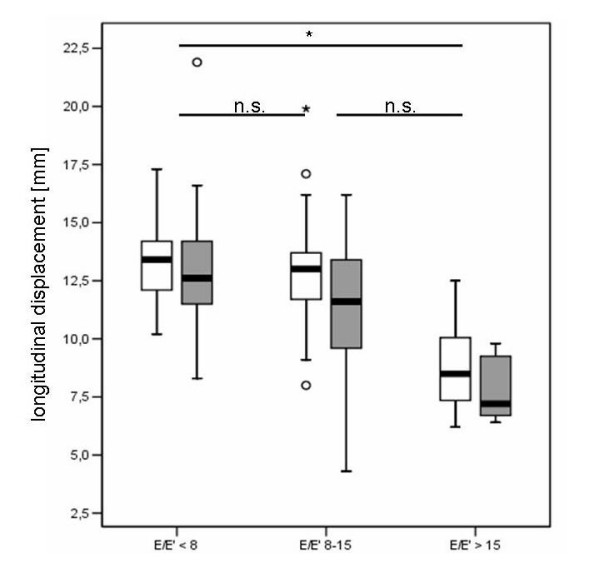
**Longitudinal displacement in patients with normal left ventricular function according to E/E'**. White: lateral, grey: septal longitudinal displacement.

Dividing the patients with preserved systolic LV function (groups 1 and 2) by E/E' (cut-point = 8) showed that there were significant differences in NT-proBNP levels (E/E' < 8: median NT-proBNP: 45.8, IQR: 172.6 pg/ml, E/E' > 8: 114.6 (261.7), p = 0.01). Classifying these patients by E/E' < 8, E/E' 8–15 and E/E' > 15 according to [14,33] revealed that those with increased filling pressures (E/E' > 15) had significantly elevated NT-proBNP and reduced longitudinal displacement values compared to patients with E/E' < 8. Strain, in contrast, was not significantly impaired. The patients with an E/E' 8–15 did not differ significantly in NT-proBNP levels compared to patients with E/E' < 8 or E/E' > 15 (figures 3 and 4).

A division by E/A (cut-point = 1, p = 0.34) or E'/A' (cut-point = 1, p = 0.54) was not associated with significantly different NT-proBNP levels. This indicates that increased E/E' is most closely linked to elevated NT-proBNP.

There was a correlation of peak systolic velocities (S') and longitudinal displacement with NT-proBNP throughout the spectrum of our patients (Spearman correlation coefficient was -0.578 (p < 0.0001) for longitudinal displacement and -0.605 (p < 0.001) for S').

## Discussion

The main finding of this study is that patients with diastolic and systolic myocardial dysfunction have significantly reduced basal left ventricular septal and lateral strain compared to healthy controls. Patients with normal LVEF and elevated left ventricular filling pressures (E/E' > 15) have significantly reduced longitudinal displacement and significantly elevated NT-proBNP. NT-proBNP can detect patients with a reduced left ventricular systolic function (LVEF < 55%) with a good diagnostic accuracy in accordance with previous studies [19-24].

In the patients with normal LV function and an elevated E/E' (8 – 15), the measurement of NT-proBNP does not add a significant diagnostic information. Therefore, the diagnostic accuracy of NT-proBNP to detect diastolic dysfunction in these patients is low. Our findings do not fully support the algorithm recently published consensus statement for the diagnosis of diastolic heart failure that emphasized E/E' and NT-proBNP [12].

In the grey zone of mildly elevated NT-proBNP and/or an E/E' between 8 and 15, it remains difficult to diagnose diastolic heart failure. This could be due to the weak correlation of invasively measured LA filling pressures and E/E' in this grey zone. The majority of patients with normal LVEF and invasively measured elevated LA filling pressures had an E/E' between 8 and 15 [37].

This underlines the importance of integrating multiple echocardiographic parameters and an individual interpretation by experienced cardiologists. Especially in the case of elevated NT-proBNP of non-cardiac causes (e.g. renal failure), the diagnostic work-up has to rely on echocardiography alone. In these borderline cases, invasive measurements by a conductance catheter should be considered to exclude relevant diastolic dysfunction [38].

Why is NT-proBNP not significantly elevated in diastolic dysfunction compared to healthy controls, but correlates to filling pressures (E/E')? We speculate that diastolic dysfunction is a process that includes a variety of mechanisms (LV hypertrophy, abnormal active relaxation, increased stiffness, increased filling pressures). The trigger for NT-proBNP release from cardiomyocytes is primarily wall stress, which is functionally reflected by filling pressures (E/E').

Our findings support the "single syndrome" theory of heart failure. Subtle changes of longitudinal myocardial function (reflected by Strain and longitudinal displacement) begin in diastolic heart failure and are further increased in systolic heart failure. However, the study by Yip [9] has seen a progressive decline of left ventricular long axis function (systolic peak mitral annular velocity) in patients with diastolic heart failure compared to healthy controls. We found that peak systolic velocities are not significantly reduced in diastolic dysfunction. The cut-off of a normal ejection fraction in their study was 45%. According to [30], a normal LVEF is ≥ 55%. For this reason, the results of [9] are possibly explained by inclusion bias of patients with reduced LVEF. In their study neither E/E', Strain or Tracking were measured. In concordance with our results, Dong [39] did not note a reduction of TDI systolic velocity (S') in patients with diastolic dysfunction.

The cut-off values of LVEF, NT-proBNP and E/E' need to be discussed, because the different studies have used different cut-offs. The definition of a normal LVEF > 50% in the ESC Guidelines [12] is somewhat arbitrary. Lang [30] has suggested 55% as the cut-off for a normal LVEF (used in our study). The lower threshold of 50% will automatically include patients with impairment of longitudinal function and will therefore alter the sensitivity and specificity for the detection diastolic dysfunction.

The NT-proBNP values physiologically increase with age [40]; therefore the threshold of 220 pg/ml in the Guidelines by Paulus [12] will lead to impaired diagnostic accuracy and false-positives in the elderly patients.

## Conclusion

In conclusion, we found that in patients with systolic and diastolic heart failure, E/E', NT-proBNP as well as Strain and longitudinal displacement add important incremental information for the severity estimation of heart failure. In patients with isolated diastolic dysfunction, Strain is significantly reduced and with increased fillings pressures longitudinal displacement is impaired, paralleled by an increase of NT-proBNP. But in a substantial subset of patients with borderline NT-proBNP and E/E', an individual analysis of all available data has to be performed.

## Limitations

We have excluded patients with atrial fibrillation because of the difficulty to assess certain diastolic function parameters (trans-mitral E/A and myocardial E'/A'). We have only measured NT-proBNP and not BNP, because recent head-to-head studies found that BNP and NT-proBNP can be used comparably [41]. We have not classified diastolic dysfunction according to restrictive, pseudo-normal or impaired relaxation. But previous studies have shown that NT-proBNP is strongly elevated in patients with pseudo-normal and restrictive filling patterns [19]. In the group of patients with normal LVEF, there were no patients with pseudo-normalization or restrictive diastolic dysfunction. No follow-up of the patients or invasive measurements were performed.

## Abbreviations

NT-proBNP: N-terminal-pro-Brain Natriuretic Peptide; PPV: Positive Predictive Value; NPV: Negative Predictive Value; ROC: Receiver Operator Characteristic; LVMI: Left Ventricular Mass Index; BMI: Body Mass Index.

## Competing interests

The authors declare that they have no competing interests.

## Authors' contributions

FK, SE, MB and ACB have designed the study and have acquired the data. SS, VR, SE, IS, MB participated in contributions to conception and, or analysis and interpretation of data. FK has written the manuscript. GB has supervised and commented the study. ACB was the supervisor of echo examinations, is head of the echo lab, contributed by revising the manuscript critically. All authors read and approved the final manuscript.

## Supplementary Material

Additional file 1 TDI image of the interventricular septum. Acquisition of a TDI image in the apical four chamber view. Click here for file

Additional file 2 How to perform the TDI analysis. Illustration of the correct acquisition a TDI region of interest (= ROI) in the basal left-ventricular septum. The size of the ROI is adjusted to the septal diameter and then, the ROI is traced manually in each frame to avoid artifacts. This acquisition applies to Velocity, Strain Rate and Displacement. Click here for file
